# Revised Body Mass Estimates for Extinct Lemurs

**DOI:** 10.1002/ajpa.70158

**Published:** 2025-11-18

**Authors:** Katharine E. T. Thompson, Ryan S. Rothman, John D. Polk, F. Tré Lawrence, William Jungers, Gabrielle A. Russo

**Affiliations:** ^1^ Interdepartmental Doctoral Program in Anthropological Sciences Stony Brook University Stony Brook New York USA; ^2^ Department of Anthropology University at Albany Albany New York USA; ^3^ Department of Anthropology Stony Brook University Stony Brook New York USA; ^4^ Department of Anatomical Sciences Stony Brook University School of Medicine Stony Brook New York USA; ^5^ Association Vahatra Antananarivo Madagascar

**Keywords:** body mass estimates, madagascar, morphometrics, primate evolution, subfossil, subfossil lemurs

## Abstract

**Objectives:**

Body mass estimates for extinct animals are critical for informing hypotheses and analyses related to behavioral ecology, extinction risk, and locomotor modes. These estimates underpin reconstructions of behavioral ecology, especially for Madagascar's extinct subfossil lemurs. Previous estimates, based on femoral and humeral midshaft cortical areas, did not account for phylogenetic relatedness, potentially impacting their accuracy. This study updates body mass estimates for extinct lemurs using phylogenetically informed methods.

**Materials and Methods:**

We analyzed 64 femora from 10 extinct lemur species. Each specimen was scanned using a Bruker SkyScan 1178 micro‐CT scanner to obtain high‐resolution images of femoral cortical areas. These data were combined to form a dataset comprising more than 125 subfossil lemur specimens across 15 identifiable species. Phylogenetically informed regression models (pGLS) incorporating femoral cortical surface area (FCSA) and femoral length (FL) as predictors were applied. Model fits were evaluated using Akaike information criterion (AIC) and adjusted *R* ^2^ values to determine the optimal predictors of body mass (BM).

**Results:**

Natural log‐transformed FCSA emerged as the best predictor of natural log‐transformed BM among living primates. This pGLS regression equation was used to estimate body mass and lower and upper 95% prediction limits for all subfossil specimens, and weighted average BM estimates were obtained for each species. Our updated body mass estimates are consistently smaller than those previously reported.

**Discussion:**

These estimates provide a more accurate basis for understanding extinct lemur life history traits, morphometrics, and ecological adaptations. These findings underscore the importance of incorporating evolutionary context in paleontological and ecological research.

## 1 Introduction

As recently as the late Holocene, at least 17 species of now‐extinct lemurs occupied Madagascar (Burney et al. 2004; Godfrey et al. 2010). In fact, all species except for *Babakotia radofilai* have been securely dated into the range of human occupation of Madagascar (Burney et al. 2004; Crowley 2010). These extinct taxa are commonly referred to as the “giant” subfossil lemurs (Godfrey et al. 1995, 2013). However, predictions of their body masses (BMs) have varied widely in the literature depending on the reference samples used (e.g., comparisons with modern lemurs vs. other primates or broader mammalian groups), the predictor variables selected (e.g., tooth size, skull length, joint size, etc.), and consideration of species relatedness (i.e., phylogenetic nonindependence among data points) (e.g., Godfrey et al. 1995; Scott et al. 2009). For example, in a review paper on the topic, Jungers et al. (2008) noted that cranial dimensions for *Megaladapis edwardsi* yield a BM estimate of 390 kg (Jungers et al. 2008; Martin 1990), maxillary first molar size yields an estimate of 156.5 kg (Gingerich et al. 1982), skeletal trunk length a range of 50–100 kg (Jungers 1978), and femoral head diameter an estimate of 67.5 kg and femoral cortical area an estimate of 85.1 kg (Jungers et al. 2008). Thus, the difference between the smallest (67.5 kg, Jungers et al. 2008) and largest (390 kg, Martin 1990) BM estimates for this taxon is nearly sixfold.

To offer more precise predictions given this breadth of BM estimates, Jungers et al. (2008) expanded on this earlier work by further using humeral and femoral cortical area at midshaft (to represent both fore‐ and hind‐limb elements) of both extant primate and nonprimate (e.g., carnivores, rodents, sloths, and koalas) mammals, to generate BM estimates for 15 subfossil lemur taxa. Megaladapids, including *Megaladapis madagascariensis*, *M. grandidieri* , and *M. edwardsi* , which were previously qualitatively described as “koala‐like” in their arboreal behavior and comparable to the size of extant calves, bear cubs, and Saint Bernard dogs (Kurtén 1971; Lavauden 1931; Simons 1972), were predicted to weigh 46.5, 74.3, and 85.1 kg, respectively. Estimates for various palaeopropithecids, a sister group of living indriids described as “sloth‐like” suspensory primates (Godfrey and Jungers 2003; Herrera and Dávalos 2016; Karanth et al. 2005; Kistler et al. 2015) were 45.8 kg for *Paleopropithecus maximus*, 41.5 kg for *Paleopropithecus ingens*, 20.7 kg for *Babakotia radofilai*, 161.2 kg for *Archaeoindris fontoynontii*, and 11.3 kg for *Mesopropithecus globiceps*. Estimates for various *archaeolemur*ids, which were likely semiterrestrial quadrupeds and are frequently compared to the body sizes of some baboons and macaques (Godfrey et al. 2006; Godfrey and Jungers 2002), included 26.5 kg for *Archaeolemur edwardsi*, 18.2 kg for *Archaeolemur majori*, and 35.4 kg for *Hadropithecus stenognathus*. Two *Pachylemur* species, which are considered larger‐bodied extinct relatives of modern *Varecia* that were also likely arboreal quadrupedal frugivores—not unlike their extant counterparts—(Godfrey et al. 2010) may have weighed roughly three times as much as modern *Varecia* species (e.g., *Varecia variegata* : ~3.5 kg; Smith and Jungers 1997). *P. insignis* , for example, has been estimated to have weighed roughly 11.5 kg (Jungers et al. 2008). Similarly, *Daubentonia robusta* (13.5 kg) and *Archaeolemur majori* (13.9 kg) were likely two of the smallest extinct lemurs (Godfrey et al. 2010), yet both were at least twice the size of the largest extant lemur species in Madagascar (e.g., *Indri indri:* 5.83 kg [M] and 6.84 kg [F], and *Propithecus diadema* [5.94 [M] to 6.26 [F]]; Smith and Jungers 1997). It should be noted that BM for these large lemurs has shown a high degree of population‐specific variation, with *Propithecus diadema* weighing as little as 4.2 kg in Tsinjoarivo (Irwin et al. 2019), to as much as 7.3 kg in Mantadia (Glander and Powzyk 1998). Zaonarivelo et al. (2007) documented BMs over 7 kg and up to 8.1 kg for *Indri indri* , and Glander and Powzyk (1998) have suggested that the designation of largest lemur may be jointly attributed to *Propithecus diadema* and *Indri indri* .

Though almost two decades old, the study by Jungers et al. (2008) remains the most recent comprehensive assessment of body sizes in subfossil lemurs. However, these estimates did not account for the impact of species relatedness, which is known to violate the assumption of independence of data points which thereby can lead to biased BM estimations (Nunn 2011). Specifically, closely related primates are more likely to have similar morphological measurements and therefore BM estimates (Nunn 2011). In this way, estimations of dependent variables of closely related species violate the assumptions of other regression models. For example, ordinary least squares (OLS) models that don't account for species relatedness assume that data points are independent and that the residuals are likewise unassociated, and the inclusion of closely related taxa breaks these assumptions. Phylogenetically informed regression methods (e.g., PGLS) account for the effects of phylogeny and thus provide more accurate and appropriate regressions for morphometrics such as BM as it varies among related taxa (Martins and Hansen 1997). Additionally, the cross‐sectional properties in the Jungers et al. (2008) predictor dataset were calculated from biplanar X‐rays using an elliptical model rather than from CT scans; the latter allows for more standardization in data collection (e.g., ensuring the cross‐section is perpendicular to the long axis of the bone) and thus presumably accurate shape measurements. More accurate estimates of subfossil lemur body size are important for reconstructing Madagascar's paleoecology and refining our understanding of extinct taxa's life histories, ecological roles, and extinction dynamics. Larger body size (which is often correlated with extended gestation and developmental periods) has been cited in support of the overkill hypothesis; this posits that human arrival and wildlife predation initiated rapid declines in subfossil lemur populations within the last ~2000 years (Peters 1986; Schollmeyer and Driver 2012). While simultaneous anthropogenic pressures undoubtedly altered Madagascar's ecosystems following human colonization, the extinction trajectories of subfossil lemurs (and other Malagasy taxa) were likely shaped by a complex array of factors. Revised BM estimates prompt renewed consideration of additional ecological forces, such as interspecific competition and natural predation, as potential co‐drivers alongside human exploitation in the disappearance of eight endemic primate genera.

## 2 Objectives

The goal of our study was to build on the work of Jungers et al. (2008) by incorporating updated phylogenies to inform comparative approaches and generate revised BM estimates for extinct subfossil lemurs. To achieve this aim, we collected microCT scans of 64 femora from 10 extinct lemur species, and examined the use of femoral cortical area and femoral length (FL) as predictors of BM using a comparative primate sample. These updated estimates will inform future inferences on subfossil lemur ecology, life history, and hypotheses concerning their extinction.

## 3 Materials and Methods

### 3.1 Sample and Data Collection

The subfossil lemur skeletal material examined in this study is housed in collections of the Department of Biological Anthropology and Paleontology at the University of Antananarivo, Madagascar and included 64 femora from *Archaeolemur edwardsi*, *Archaeolemur majori*, *Megaladapis madagascariensis*, *Megaladapis grandidieri*, *Megaladapis edwardsi*, *Mesopropithecus globiceps*, *Mesopropithecus pithecoides*, *Pachylemur insignis*, *Paleopropithecus ingens*, and *Paleopropithecus maximus* (Table 1). Bones were transported with permission to the Centre ValBio (CVB) field research station based in Ranomafana (Rothman et al. 2022). All subfossil lemur specimens were scanned at CVB using a Bruker SkyScan 1178 Micro‐CT scanner. Scans were conducted with the following parameters: a source voltage of 65 kV and source current of 615 μA, paired with a 0.5 mm aluminum filter to reduce beam hardening artifacts. The digital camera resolution was set to 1280 × 1024 pixels, yielding an image pixel size of 83.7 μm. Scans followed a step‐and‐shoot motion with a rotation step of 0.54° over a total angular range of 203.58°. Each scan used an exposure time of 720 ms with 12‐frame averaging to enhance image quality. Reconstruction was performed in NRecon software, applying a Hamming filter for optimal clarity, with ring artifact correction set to 10 and a beam hardening correction of 50%. This setup provided high‐resolution imaging while minimizing artifacts, making it ideal for capturing the fine structural details of subfossil specimens up to 200 mm in length. The SkyScan‐1178 is a high‐speed micro‐CT scanner with dual camera‐source configurations, 20–65 kV X‐ray sources, and 1280 × 1024 pixel digital cameras, providing an 82 mm field‐of‐view and 80–160 μm resolution, ideal for imaging small specimens up to 200 mm in length (Liu et al. 2008).

**TABLE 1:** Species and sample sizes of femora collected from the University of Antananarivo's collections representing the original data collection of this study (*N* = 64 specimens).

Genus	Species	Sample size
*Archaeolemur*	*edwardsi*	6
*Archaeolemur*	*majori*	14
Megaladapis	*edwardsi*	6
Megaladapis	*grandidieri*	1
Megaladapis	*madagascariensis*	2
Mesopropithecus	*globiceps*	3
Mesopropithecus	*pithecoides*	1
Pachylemur	*insignis*	25
Palaeopropithecus	*ingens*	1
Palaeopropithecus	*maximus*	5
	Total	64

**FIGURE 1:**
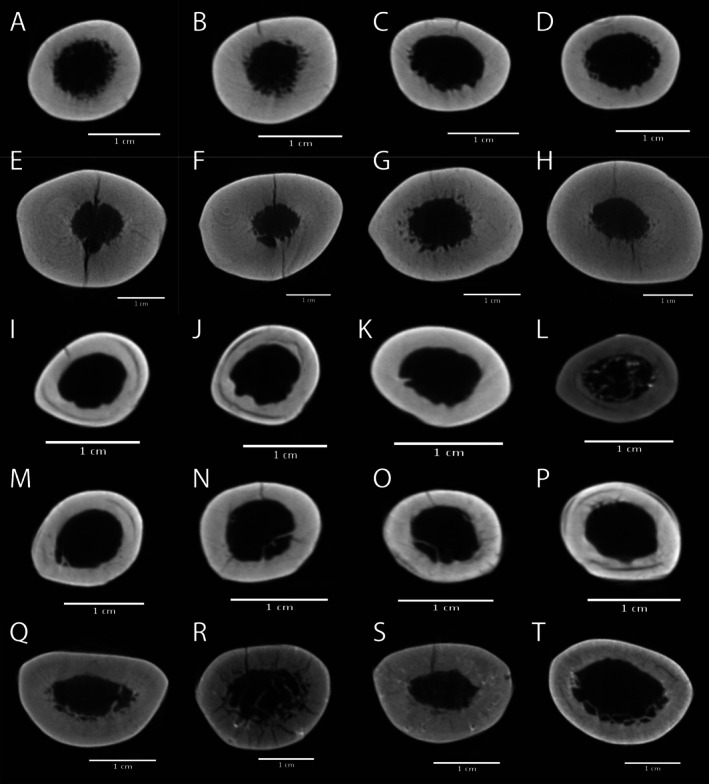
Midshaft (50%) femoral cross‐sections of subfossil lemurs from five genera: *Archaeolemur* (A–D), *Megaladapis* (E–H), *Mesopropithecus* (I–L), *Pachylemur* (M–P), and *Paleopropithecus* (Q–T). Cross‐sections reveal cortical area distribution used for body mass estimation. Scale bars represent 10 mm.

Prior to scanning, maximum bone lengths (in mm) were measured using an osteometric board and the shaft midpoint (i.e., 50% of total length) was marked with a rubber marker detectable on scan images. Image stacks were imported into NRecon for refinement and reconstruction. FCSA (mm^2^) was extracted using the BoneJ plug‐in within ImageJ 32 for each specimen (Doube et al. 2010). Some specimens were determined to be beyond the size limitations of the scanner bed and were therefore excluded, as were software‐determined outliers (*n* = 1) and scans in which part of the specimen was obscured from the scanner's camera (*n* = 14). The final sample contained 64 specimens representing 10 species (Figure 1).

**FIGURE 2:**
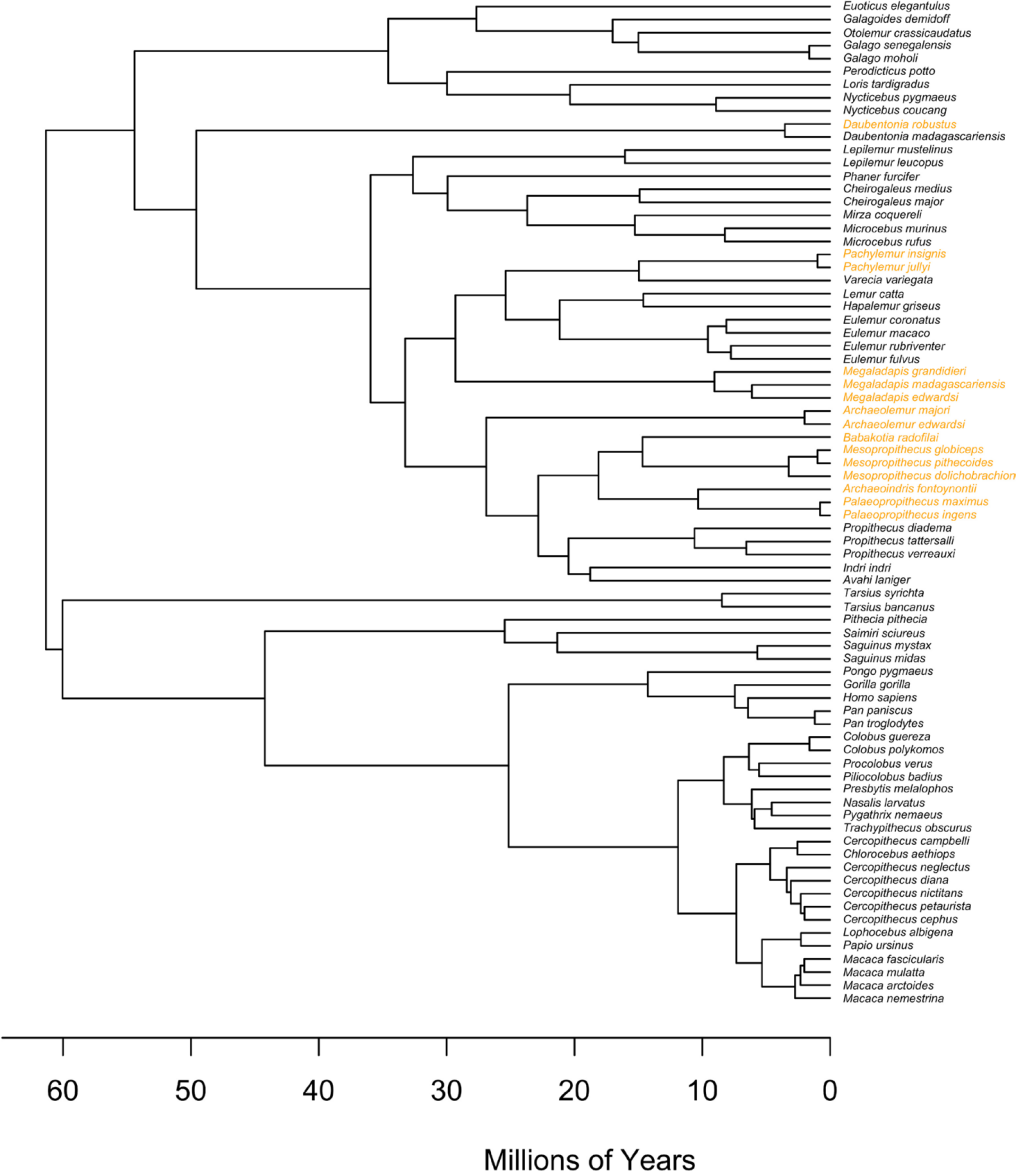
Phylogenetic tree of 77 primate species, reflecting the taxa included in the study. Based on the strepsirrhine phylogeny by Herrera and Dávalos (2016) with species placements by Kistler et al. (2015), this tree incorporates genetic and morphological data. Scale bar represents evolutionary divergence time in millions of years. Extant taxa (*N* = 62) are displayed with black tip labels, while subfossil taxa (*N* = 15) are displayed with orange tip labels. Scale bar represents evolutionary divergence time in millions of years.

The extant primate long bone data set came from Polk et al. (2000), which, in addition to generating new data, compiled primate data from multiple sources—primarily (Demes and Jungers 1993; Runestad 1994) (see Table S1). Additional supplemental data were provided by Brigitte Demes, and Kristian Carlson, and some BM data were sourced from Smaers et al. (2021). Primate data were obtained from wild‐shot individuals with a few exceptions: *Lemur catta* , *Mirza*, *Propithecus tattersalli* (see Demes and Jungers 1993 for discussion), *Daubentonia* which died shortly after capture, two individuals of *Cebuella* that lived in seminatural captivity (Runestad 1994), and *Macaca mulatta* (Burr et al. 1989). BM was obtained from museum records for wild‐shot individuals for all cercopithecoids and hominoids that were newly published in Polk et al. (2000). For the remainder, species‐mean BMs were obtained from published records (also see references in Demes and Jungers 1993; Runestad 1994, 1997; Smith and Jungers 1997). Methods for quantifying cross‐sectional properties varied among and within studies. For data collected by Polk et al., CT scans and physical sections were manually digitized using SLICE (Nagurka and Hayes 1980) have s, while source data for platyrrhines and strepsirrhines were orthogonally‐oriented biplanar radiographs assuming a symmetrically placed marrow cavity (Demes and Jungers 1993; Runestad 1994). Since each of these methods yields an accurate approximation of the cross‐sectional properties for each specimen (Biknevicius and Ruff 1992; Ruff 1989), and there are no systematic differences in accuracy among the methods, the data are combined in this study. For further details on the collection of these data see Polk et al. (2000) and the source publications (Demes and Jungers 1993; Runestad 1994). Specimens with incomplete data on study variables or with taxonomic identifications that could not be verified were omitted leaving a final reference sample of 570 individual primates, for a total of 77 species means (Figure 2).

## 4 Analysis

A phylogenetic tree was constructed by integrating Herrera and Dávalos's (2016) strepsirrhine phylogeny, incorporating the placement of *Megaladapis* from Kistler et al. (2015); this employed a total evidence approach combining genetic and morphological data, with a broader primate phylogeny from the *10kTrees* project (Arnold et al. 2010), an online resource providing divergence estimates across primates. Adjustments for divergence times were refined using Springer et al. (2012), whose species supermatrix captures macroevolutionary dynamics and historical biogeography across primate lineages. We resolved all polytomies from the tree and then readjusted the tree to reflect our subset of 62 extant primates (Figure S1).

All analyses were performed in the R statistical environment Version 4.1.3 (R Core Team 2023). We performed two univariate and one multivariate phylogenetic generalized least squares (PGLS) regression using the caper package in R in a log‐natural space (Orme 2013), with FCSA (mm^2^) and FL (mm) as predictor variables, and BM (kg) as the response variable. PGLS is a common technique in the estimation of BM for extinct taxa using extant data (Wright et al. 2024). These regressions allow for heteroscedasticity and serial correlations among related species which would defy the assumptions of a classic linear model by including an error term that incorporates species relatedness (Martins and Hansen 1997). When a subset of species within the data is more closely related to itself than the rest of the dataset, the cluster may pull the regression line toward itself and bias resulting predictions. PGLS can account for this species‐relatedness by downweighting closely related clades according to phylogenetic distance and shared branch lengths (Martins and Hansen 1997).

**FIGURE 3:**
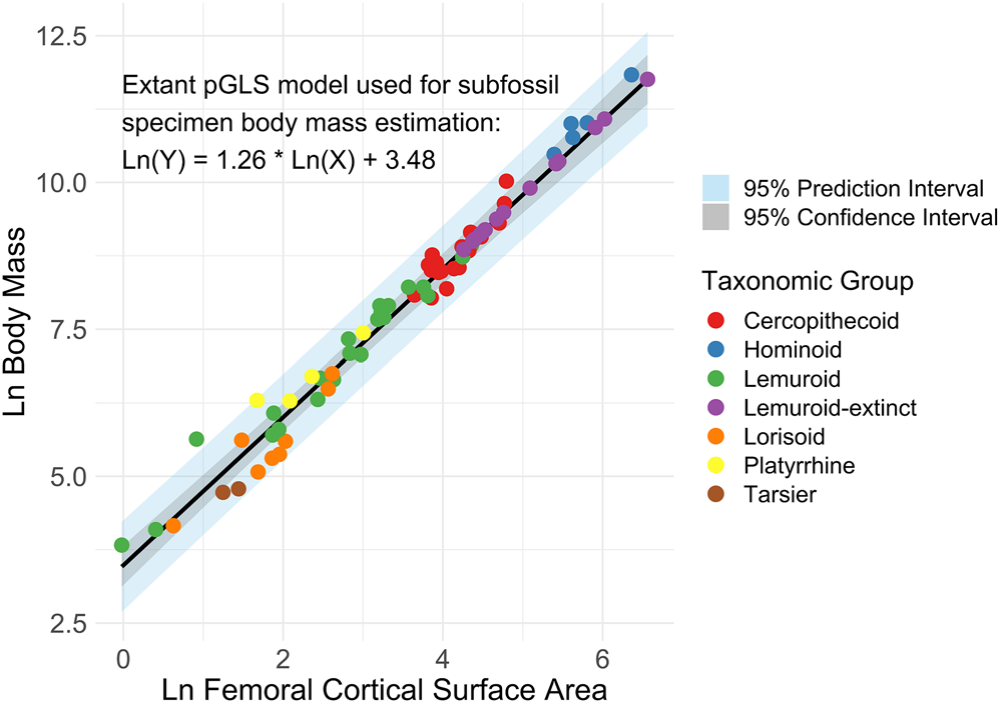
Scatterplot characterizing natural log‐transformed body mass (kg) regressed against natural log‐transformed femoral cortical surface area (FCSA, mm^2^) across our sample of 62 extant primates for which the phylogenetically informed slope and 95% confidence and prediction intervals are drawn. An additional 15 extinct lemur species are displayed as estimated by the extant regression. Data points are color‐coded by taxonomic group.

The best fit among these three candidate models was determined by comparing Akaike information criterion (AIC) scores, with the lowest score indicating the best fit model for the relationship among the variables being regressed, as well as the adjusted *R* ^2^ which indicates the amount of variance in BM explained by each predictor. To generate BM estimates for subfossil specimens, we used the PGLS model of best fit to predict natural log‐transformed values from observed femoral measurements and calculated 95% phylogenetic prediction intervals for each specimen by propagating uncertainty via a variance–covariance matrix scaled by Pagel's *λ* (Pagel 1999) estimated from the extant primate data. We then calculated 95% confidence intervals for the regression line, incorporating phylogenetic nonindependence through the same *λ*‐scaled extant variance–covariance matrix (Figure 3). Subfossil data was then averaged across specimens, accounting for the weight of how many specimens were included for each species.

## 5 Results

Results for univariate and multivariate regression models, including model structure, regression equation, phylogenetic signal, standard error and *p*‐value associated with each predictor variable, AIC value, and adjusted *R* ^2^ are presented in Table 2. The first univariate PGLS model (Model 1) to predict BM in kg, based on FCSA, was significant (*F*_1,60_ = 771.2, *p* < 0.001), with an adjusted *R* ^2^ of 0.93. In this regression, BM increases 1.26 natural logged units (95% CI: 1.17–1.35) for each natural logged‐unit increase in FCSA. Phylogenetic signal setting Pagel's lambda to “Maximum Likelihood” was found to be 0.64.

**TABLE 2:** Model summary information across the three tested phylogenetic generalized least‐squares (pGLS) regressions including the regression equation, Pagel's lambda (*λ*) indicating phylogenetic signal, standard errors and *p*‐values associated with each predictor variable, Akaike's information criterion (AIC), and adjusted *R* ^2^ value. Model 1 predicts natural‐logged (ln) body mass (BM; kg) using ln femoral cortical surface area (FCSA; mm^2^), Model 2 predicts ln BM using ln femoral length (FL; mm), and multivariate Model 3 predicts ln BM using both ln FCSA and ln FL.

Model	Regression equation	Pagel's *λ*	SE	*p*	AIC	Adj. *R* ^2^
1. BM ~ FCSA	ln(*Y*) = 1.26 × ln(*X*) + 3.48	0.64	0.05	< 0.001	18	93%
2. BM ~ FL	ln(*Y*) = 2.65 × ln(*X*) − 5.19	0.88	0.16	< 0.001	56	82%
3. BM ~ FCSA + FL	ln(*Y*) = 1.17 × ln(*X*1) + 0.21 × ln(X2) + 2.79	0.69	0.16 (FCSA); 0.34 (FL)	< 0.001 (FCSA); 0.55 (FL)	20	92%

The second univariate PGLS model to predict BM (Model 2), based on FL, was also significant (*F*_1,60_ = 276.6, *p* < 0.001), with an adjusted *R* ^2^ of 0.82. In this regression, BM increases 2.65 natural logged units (95% CI: 2.33–2.97) for each natural logged‐unit increase in FL. Phylogenetic signal setting Pagel's lambda to “Maximum Likelihood” was found to be 0.88.

The third PGLS using both FCSA and FL as predictors of BM (Model 3) was also significant (*F*_2,59_ = 357.9, *p* < 0.001), with an adjusted *R* ^2^ of 0.92. In this regression, BM increases 1.17 natural logged units (95% CI: 0.86–1.48) for each natural logged unit of FCSA, and 0.21 natural logged units (95% CI: −0.48 to 0.90) for each natural logged unit of FL. In this multivariate regression, only FCSA was a significant predictor of BM (refer to Table 2). Phylogenetic signal setting Pagel's lambda to “Maximum Likelihood” was found to be 0.69.

Model 1 (AIC = 18) was found to be the best‐fit regression of the three tested models, explaining most of the variation observed in BM with the fewest number of predictors. Model 2 (AIC = 56), while possessing a significant association, explained the least amount of variation in BM (adj. *R* ^2^ = 0.82). In Model 3 (AIC = 20), only FCSA was significant (*p* < 0.001); FL was not found to be significant (*p* = 0.55). Therefore, FCSA explains 93% of the variation in BM, a very strong predictor, and with moderate phylogenetic signal (*λ* = 0.64).

The PGLS regression using the natural log of FCSA to predict the natural log of BM (Model 1) therefore was used to generate new BM estimations for the subfossil specimens including lower and upper phylogenetically informed 95% prediction limits (refer to Table 3). Weighted means combining the univariate regression estimates (including lower and upper 95% prediction limits) using specimen data both from this study (*nT* = 64 specimens) and Jungers et al. (2008; *nJ* = 63 specimens) (Table 4) place *Archaeolemur edwardsi* at 20.01 (9.36, 42.78) kg, *Archaeolemur majori* at 13.2 (6.22, 27.99) kg, *Megaladapis edwardsi* at 64.97 (29.53, 142.91) kg, *Megaladapis grandidieri* at 56.1 (25.6, 122.91) kg, *Megaladapis madagascariensis* at 31.56 (14.61, 68.16) kg, *Mesopropithecus globiceps* at 7.02 (3.34, 14.73) kg, *Mesopropithecus pithecoides* at 9.14 (4.34, 19.26) kg, *Mesopropithecus dolicobrachion* at 8.09 (3.84, 17.01) kg, *Palaeopropithecus ingens* at 30.32 (14.04, 65.46) kg, *Paleopropithecus maximus* at 30.29 (14.04, 65.34) kg, *Babakotia radofilai* at 11.8 (5.57, 24.96) kg, *Archaeoindris fontoynontii* at 127.74 (56.95, 286.52) kg, *Pachylemur insignis* at 8.33 (3.96, 17.53) kg, *Pachylemur jullyi* at 9.75 (4.62, 20.58) kg, and *Daubentonia robusta* at 9.84 (4.66, 20.76) kg. Two femoral specimens of *Archaeolemur* which were not identified to the species level by Jungers et al. (2008) place at 16.85 (7.91, 35.9) and 16.54 (7.77, 35.22) kg, respectively (Table 3), or averaged together at 16.7 (7.84, 35.56) kg (Table 4). See Figure 3 for a visualization of these subfossil BM estimates (with confidence and prediction intervals) in the context of our best‐fit PGLS regression using FCSA. Our updated BM estimates for subfossil lemur species from specimen weighted means are significantly lower than those published in Jungers et al. (2008; paired *t*‐test: *t* = −13.6, df = 13, *p* < 0.001) and Godfrey et al. (1995; *t* = −6.5, df = 13, *p* < 0.001). Refer to Table 5 for BM estimate comparisons between these studies.

**TABLE 3:** Subfossil lemur femoral cortical surface area measurements (FCSA; mm^2^), body mass estimates (BM; kg) and lower and upper 95% prediction limits for each unique subfossil specimen included in this study (*N* = 127). Information on specimen unique ID and dataset (original data; Jungers et al. 2008) is included.

Specimen	ID	DataSet	FCSA (mm^2^)	Estimate (kg)	Lower 95 kg	Upper 95 kg
*Archaeoindris fontoynontii*	L1	Jungers	708.10	127.74	56.95	286.52
*Archaeolemur sp*.	AN3	Jungers	142.01	16.85	7.91	35.90
*Archaeolemur sp*.	AN4	Jungers	139.91	16.54	7.77	35.22
*Archaeolemur edwardsi*	AB	Thompson	162.80	20.02	9.37	42.79
*Archaeolemur edwardsi*	AF	Thompson	180.30	22.77	10.63	48.80
*Archaeolemur edwardsi*	AH	Thompson	178.20	22.44	10.47	48.07
*Archaeolemur edwardsi*	BD	Thompson	160.80	19.71	9.23	42.12
*Archaeolemur edwardsi*	BG	Thompson	123.40	14.12	6.65	29.97
*Archaeolemur edwardsi*	BH	Thompson	148.00	17.76	8.33	37.86
*Archaeolemur edwardsi*	AE8	Jungers	168.02	20.83	9.74	44.57
*Archaeolemur edwardsi*	AE9	Jungers	147.15	17.63	8.27	37.58
*Archaeolemur edwardsi*	AE10	Jungers	175.65	22.03	10.29	47.19
*Archaeolemur edwardsi*	AE11	Jungers	170.41	21.21	9.91	45.38
*Archaeolemur edwardsi*	AE12	Jungers	163.40	20.12	9.41	43.00
*Archaeolemur edwardsi*	AE13	Jungers	173.64	21.72	10.14	46.49
*Archaeolemur edwardsi*	AE14	Jungers	168.38	20.89	9.77	44.69
*Archaeolemur edwardsi*	AE15	Jungers	155.58	18.91	8.86	40.37
*Archaeolemur majori*	AA	Thompson	120.80	13.75	6.48	29.16
*Archaeolemur majori*	AC	Thompson	136.40	16.02	7.53	34.09
*Archaeolemur majori*	AD	Thompson	93.40	9.94	4.71	20.97
*Archaeolemur majori*	AG	Thompson	141.00	16.70	7.84	35.57
*Archaeolemur majori*	AI	Thompson	122.30	13.96	6.58	29.63
*Archaeolemur majori*	AK	Thompson	90.70	9.58	4.54	20.20
*Archaeolemur majori*	AM	Thompson	108.00	11.94	5.64	25.26
*Archaeolemur majori*	AN	Thompson	128.40	14.84	6.99	31.54
*Archaeolemur majori*	AR	Thompson	100.90	10.95	5.18	23.15
*Archaeolemur majori*	AT	Thompson	109.20	12.10	5.72	25.62
*Archaeolemur majori*	AV	Thompson	124.30	14.25	6.71	30.25
*Archaeolemur majori*	AW	Thompson	116.40	13.12	6.19	27.81
*Archaeolemur majori*	BC	Thompson	119.40	13.54	6.39	28.73
*Archaeolemur majori*	BR	Thompson	120.00	13.63	6.43	28.92
*Archaeolemur majori*	AM3	Jungers	134.10	15.68	7.37	33.35
*Archaeolemur majori*	AM4	Jungers	110.90	12.34	5.83	26.13
*Archaeolemur majori*	AM5	Jungers	108.82	12.05	5.69	25.51
*Babakotia radofilai*	B5	Jungers	112.31	12.54	5.92	26.56
*Babakotia radofilai*	B6	Jungers	94.79	10.13	4.80	21.37
*Babakotia radofilai*	B7	Jungers	106.50	11.73	5.54	24.81
*Babakotia radofilai*	B8	Jungers	114.09	12.79	6.04	27.10
*Daubentonia robusta*	DR4	Jungers	92.44	9.81	4.65	20.69
*Daubentonia robusta*	DR5	Jungers	92.87	9.87	4.68	20.82
*Megaladapis edwardsi*	NC	Thompson	333.70	49.48	22.67	108.01
*Megaladapis edwardsi*	NE	Thompson	449.80	72.09	32.69	158.98
*Megaladapis edwardsi*	NF	Thompson	415.80	65.29	29.69	143.59
*Megaladapis edwardsi*	NG	Thompson	436.50	69.42	31.51	152.91
*Megaladapis edwardsi*	NH	Thompson	299.10	43.10	19.82	93.76
*Megaladapis edwardsi*	NI	Thompson	398.40	61.87	28.17	135.85
*Megaladapis edwardsi*	ME7	Jungers	469.75	76.15	34.48	168.18
*Megaladapis edwardsi*	ME8	Jungers	446.91	71.51	32.44	157.66
*Megaladapis edwardsi*	ME9	Jungers	448.67	71.87	32.59	158.46
*Megaladapis edwardsi*	ME10	Jungers	469.52	76.10	34.46	168.07
*Megaladapis edwardsi*	ME11	Jungers	381.38	58.55	26.71	128.39
*Megaladapis edwardsi*	ME12	Jungers	410.04	64.15	29.19	141.01
*Megaladapis grandidieri*	ND	Thompson	381.00	58.48	26.67	128.22
*Megaladapis grandidieri*	MGr3	Jungers	417.78	65.68	29.86	144.47
*Megaladapis grandidieri*	MGr4	Jungers	359.91	54.43	24.87	119.11
*Megaladapis grandidieri*	MGr5	Jungers	291.02	41.64	19.16	90.50
*Megaladapis grandidieri*	MGr6	Jungers	328.48	48.51	22.23	105.83
*Megaladapis grandidieri*	MGr7	Jungers	428.61	67.84	30.82	149.34
*Megaladapis madagascariensis*	NA	Thompson	233.00	31.46	14.58	67.91
*Megaladapis madagascariensis*	NB	Thompson	190.80	24.46	11.39	52.49
*Megaladapis madagascariensis*	MM5	Jungers	207.00	27.10	12.60	58.30
*Megaladapis madagascariensis*	MM6	Jungers	217.32	28.82	13.38	62.08
*Megaladapis madagascariensis*	MM7	Jungers	275.31	38.83	17.90	84.23
*Megaladapis madagascariensis*	MM8	Jungers	282.71	40.15	18.49	87.17
*Megaladapis madagascariensis*	MM9	Jungers	251.12	34.58	15.98	74.80
*Megaladapis madagascariensis*	MM10	Jungers	207.03	27.11	12.60	58.31
*Mesopropithecus dolichobrachion*	MD2	Jungers	79.31	8.09	3.84	17.01
*Mesopropithecus globiceps*	MA	Thompson	63.20	6.07	2.90	12.73
*Mesopropithecus globiceps*	MB	Thompson	70.60	6.98	3.33	14.66
*Mesopropithecus globiceps*	MC	Thompson	72.10	7.17	3.42	15.06
*Mesopropithecus globiceps*	MG6	Jungers	74.54	7.48	3.56	15.71
*Mesopropithecus globiceps*	MG7	Jungers	58.74	5.54	2.65	11.59
*Mesopropithecus globiceps*	MG8	Jungers	78.57	7.99	3.80	16.81
*Mesopropithecus globiceps*	MG9	Jungers	75.67	7.62	3.63	16.02
*Mesopropithecus globiceps*	MG10	Jungers	64.71	6.26	2.99	13.12
*Mesopropithecus globiceps*	MG11	Jungers	78.98	8.04	3.82	16.92
*Mesopropithecus pithecoides*	MD	Thompson	87.40	9.14	4.34	19.26
*Pachylemur insignis*	DA	Thompson	89.40	9.40	4.46	19.83
*Pachylemur insignis*	DB	Thompson	85.00	8.83	4.19	18.59
*Pachylemur insignis*	DC	Thompson	72.40	7.21	3.43	15.14
*Pachylemur insignis*	DD	Thompson	103.00	11.24	5.32	23.77
*Pachylemur insignis*	DE	Thompson	84.20	8.72	4.14	18.36
*Pachylemur insignis*	EA	Thompson	77.00	7.79	3.71	16.38
*Pachylemur insignis*	EB	Thompson	84.90	8.81	4.18	18.56
*Pachylemur insignis*	EC	Thompson	79.50	8.11	3.86	17.06
*Pachylemur insignis*	ED	Thompson	78.60	8.00	3.80	16.82
*Pachylemur insignis*	EE	Thompson	71.90	7.15	3.40	15.00
*Pachylemur insignis*	EF	Thompson	75.00	7.54	3.59	15.84
*Pachylemur insignis*	EG	Thompson	79.70	8.14	3.87	17.12
*Pachylemur insignis*	EH	Thompson	75.60	7.61	3.62	16.00
*Pachylemur insignis*	EI	Thompson	87.90	9.21	4.37	19.40
*Pachylemur insignis*	EJ	Thompson	83.30	8.60	4.09	18.11
*Pachylemur insignis*	EK	Thompson	68.90	6.77	3.23	14.21
*Pachylemur insignis*	EL	Thompson	73.40	7.33	3.49	15.41
*Pachylemur insignis*	EM	Thompson	75.70	7.63	3.63	16.03
*Pachylemur insignis*	EN	Thompson	88.60	9.30	4.41	19.60
*Pachylemur insignis*	EO	Thompson	81.00	8.30	3.95	17.47
*Pachylemur insignis*	EP	Thompson	103.20	11.27	5.33	23.83
*Pachylemur insignis*	EQ	Thompson	80.00	8.18	3.89	17.20
*Pachylemur insignis*	ER	Thompson	70.20	6.93	3.30	14.55
*Pachylemur insignis*	ES	Thompson	85.10	8.84	4.20	18.61
*Pachylemur insignis*	ET	Thompson	83.40	8.62	4.09	18.14
*Pachylemur insignis*	PIn1	Jungers	83.69	8.65	4.11	18.22
*Pachylemur insignis*	PIn2	Jungers	72.38	7.21	3.43	15.13
*Pachylemur insignis*	PIn3	Jungers	90.78	9.59	4.55	20.22
*Pachylemur insignis*	PIn4	Jungers	110.18	12.24	5.78	25.92
*Pachylemur insignis*	PIn9	Jungers	80.20	8.20	3.90	17.25
*Pachylemur insignis*	PIn10	Jungers	89.06	9.36	4.44	19.73
*Pachylemur insignis*	PIn11	Jungers	71.16	7.05	3.36	14.81
*Pachylemur insignis*	PIn17	Jungers	55.30	5.13	2.46	10.73
*Pachylemur insignis*	PIn18	Jungers	63.60	6.12	2.92	12.83
*Pachylemur jullyi*	PJ1	Jungers	92.50	9.82	4.65	20.71
*Pachylemur jullyi*	PJ2	Jungers	83.83	8.67	4.12	18.26
*Pachylemur jullyi*	PJ7	Jungers	96.64	10.37	4.91	21.91
*Pachylemur jullyi*	PJ8	Jungers	94.99	10.15	4.81	21.43
*Paleopropithecus ingens*	PI2	Jungers	275.04	38.78	17.88	84.13
*Paleopropithecus ingens*	PI5	Jungers	156.99	19.13	8.96	40.84
*Paleopropithecus ingens*	PI7	Jungers	203.40	26.51	12.33	57.00
*Paleopropithecus ingens*	CB	Thompson	264.20	36.86	17.01	79.87
*Paleopropithecus maximus*	PM4	Jungers	230.22	30.99	14.36	66.87
*Paleopropithecus maximus*	PM5	Jungers	264.29	36.88	17.02	79.91
*Paleopropithecus maximus*	PM6	Jungers	251.77	34.69	16.03	75.05
*Paleopropithecus maximus*	CA	Thompson	214.30	28.31	13.15	60.97
*Paleopropithecus maximus*	CC	Thompson	228.90	30.77	14.26	66.38
*Paleopropithecus maximus*	CD	Thompson	163.80	20.18	9.44	43.13
*Paleopropithecus maximus*	CG	Thompson	215.50	28.51	13.24	61.41
*Paleopropithecus maximus*	CH	Thompson	236.00	31.97	14.81	69.04

**TABLE 4:** Species weighted‐average subfossil lemur cortical surface area measurements (FCSA; mm^2^), body mass estimates (BM; kg), and lower and upper 95% prediction limits. Sample sizes are included showing the number of specimens for each species that came from (original data; *nT*), Jungers et al. (2008; *nJ*), and total sample size per species (*N*).

Species	*nT*	*nJ*	*N*	FCSA (mm^2^)	BM (kg)	Lower 95 kg	Upper 95 kg
*Archaeoindris fontoynontii*	0	1	1	708.10	127.74	56.95	286.52
*Archaeolemur* sp.	0	2	2	140.96	16.70	7.84	35.56
*Archaeolemur edwardsi*	6	8	14	162.55	20.01	9.36	42.78
*Archaeolemur majori*	14	3	17	116.77	13.20	6.22	27.99
*Babakotia radofilai*	0	4	4	106.92	11.80	5.57	24.96
*Daubentonia robusta*	0	2	2	92.66	9.84	4.66	20.76
*Megaladapis edwardsi*	6	6	12	413.30	64.97	29.53	142.91
*Megaladapis grandidieri*	1	5	6	367.80	56.10	25.60	122.91
*Megaladapis madagascariensis*	2	6	8	233.04	31.56	14.61	68.16
*Mesopropithecus dolichobrachion*	0	1	1	79.31	8.09	3.84	17.01
*Mesopropithecus globiceps*	3	6	9	70.79	7.02	3.34	14.73
*Mesopropithecus pithecoides*	1	0	1	87.40	9.14	4.34	19.26
*Pachylemur insignis*	25	9	34	80.98	8.33	3.96	17.53
*Pachylemur jullyi*	0	4	4	91.99	9.75	4.62	20.58
*Paleopropithecus ingens*	1	3	4	224.91	30.32	14.04	65.46
*Paleopropithecus maximus*	5	3	8	225.60	30.29	14.04	65.34

**TABLE 5:** Subfossil lemur body mass estimations (kg) compared across studies: original data, Godfrey et al. (1995), and Jungers et al. (2008). Comparisons between Thompson–Godfrey and Thompson–Jungers estimates using paired‐samples *t*‐tests.

Species	Sample: Thompson	Sample: Godfrey	Sample: Jungers
*Archaeolemur edwardsi*	20.0	24.5	26.5
*Archaeolemur majori*	13.2	13.9	18.2
*Megaladapis edwardsi*	65.0	75.4	85.1
*Megaladapis grandidieri*	56.1	63.0	74.3
*Megaladapis madagascariensis*	31.6	38.0	46.5
*Mesopropithecus globiceps*	7.0	9.4	11.3
*Mesopropithecus pithecoides*	9.1	9.7	NA
*Mesopropithecus dolichobrachion*	8.1	NA	13.7
*Palaeopropithecus ingens*	30.3	39.5	41.5
*Palaeopropithecus maximus*	30.3	52.6	45.8
*Babakotia radofilai*	11.8	16.2	20.7
*Archaeoindris fontoynontii*	127.7	197.5	161.2
*Pachylemur insignis*	8.3	10.0	11.5
*Pachylemur jullyi*	9.8	12.8	13.4
*Daubentonia robustus*	9.8	13.5	14.2

Paired *t*‐test comparison	*t*	df	*p*
Thompson vs. Godfrey	−6.5	13	9.5E−06
Thompson vs. Jungers	−13.6	13	2.3E−09

## 6 Discussion

This project reexamined the use of FL and cross‐sectional area as predictors of BM in subfossil lemurs using phylogenetically informed methods. Resulting estimates are overall smaller than previous estimates based on the same morphometrics. This suggests that the BM estimates found in the existing literature may have been inflated due to species relatedness and underscores the importance of employing phylogenetically informed methods for BM estimates. This is critical to informing interpretations about subfossil lemur life history, ecology, and hypotheses concerning their extinction.

The smaller BM estimates from our study (compared to nonphylogenetically informed prior studies) have implications for the inferred life history trends of subfossil lemurs. As several species of extinct lemurs had been estimated to have BMs comparable to gorillas, chimpanzees and orangutans, it has been suggested that they would have similarly long life spans, slow growth and development rates, late ages of first reproduction, and low reproductive rates (Burney 1999; Godfrey and Jungers 2003; Schwartz et al. 2002). Previously published BM estimates for subfossil lemurs have also been used to calculate predictions of reproductive rates, and these estimations will need to be reassessed (Godfrey et al. 2013). Additionally, such BM estimates have been evaluated in relation to other life history traits including brain volume, interbirth intervals, gestation length, and weaning age (Godfrey et al. 2013). Relative brain size calculations and theories regarding encephalization of these taxa therefore also need to be revisited considering this new data. In light of the findings from our study, careful reconsideration of these life history parameters is warranted.

Likewise, estimations of BM have played a prominent role in discussions concerning the rapid and possibly anthropogenic extinction of these species. Peters (1986) and Schollmeyer and Driver (2012) assert that large body size is a significant risk factor in anthropogenic extinction. Crowley (2010) notes that all of Madagascar's endemic species weighing greater than 10 kg are now extinct. Some authors have suggested that the large BM of species such as *Archaeoindris* constrained them to terrestriality; this may have made them easier targets of hunting activities (Cowlishaw and Dunbar 2021; Jungers et al. 2008; Orlando et al. 2008). Additionally, larger‐bodied animals with slower growth rates and longer life histories exhibit decreased resilience (Pimm 1991). Therefore, they will take longer to return to stable population levels after exposure to hunting pressure and may still be at lower abundances when subsequent pressures occur. Large body size, combined with other factors such as terrestriality and diurnality may have made certain species of subfossil lemurs particularly susceptible to overexploitation and thus anthropogenic extinction (Godfrey et al. 1997; Jernvall and Wright 1998). Our calculations indicating that subfossil lemurs may have been smaller than previously thought thus have implications for hypotheses concerning their extinction risks.

Jungers (1987) concluded that the “giant” aye‐aye (*Daubentonia robusta*) was much larger than the extant form ( *Daubentonia madagascariensis* ), and despite differences in limb proportions, specializations of the hand were quite similar (Lamberton 1934). Godfrey et al. (2016) concluded that feeding adaptations in the two species were therefore likely analogous. Given this, *Daubentonia robusta* appears to have competitively excluded *Daubentonia madagascariensis* from the southwest of the island with no evidence of subsequent colonization of this region by the extant aye–aye. Indeed it remains absent from the southwest quarter today. Our revised estimates indicating a smaller BM for *Daubentonia robusta* further support this hypothesis as these closely related taxa with similar adaptations may especially have had overlapping diets if they were also closer in BM. Comparable alimentary niches would have increased interspecific competition, and may have led to the allopatric distribution evidenced in the subfossil record.

It is possible that concomitant instances of interspecific competition could have also occurred among other ancestral communities including both extant and subfossil lemurs which were closely related and/or similarly adapted. *Pachylemur*, for example, appears to occupy a niche very similar to the extant *Varecia* (Walker 1974). This may justify a reappraisal of theories surrounding the extinction of the subfossil lemurs. With BM values for several species now closer to those of the largest extant lemurs; for example, all species of *Mesopropithecus* and *Pachylemur* are estimated to fall either within the range of *Indri indri* (up to 8.1 kg: Zaonarivelo et al. 2007) and *Propithecus diadema* (up to 7.3 kg; Glander and Powzyk 1998), or very near to their upper limits, we might ask why these species were more vulnerable to population declines than their surviving relatives, especially if interbirth intervals and developmental periods may not have been as lengthy as previously assumed. Human predation pressure, and/or habitat modification may have therefore had a compounding effect on species which were perhaps already at a competitive disadvantage.

The revised estimates may also help to illuminate the still poorly studied predator/prey interactions of the subfossil lemurs. Meador (2017) found that the largest known terrestrial Malagasy predator, *Cryptoprocta spelea* (the giant fossa) would have likely hunted lemurs weighing as much as 85 kg. *Cryptoprocta ferox* (the extant fossa) is known to hunt in groups in order to take down larger prey and it is believed that the giant fossa may have employed similar predation strategies (Goodman and Jungers 2014). Nearly all of the subfossil lemurs are estimated to fall well below the estimated limit of vulnerability to *C. spelea* hunting, and even the largest species could have been hunted cooperatively. There is evidence that predation by *C. ferox* has caused the local extirpation of *Propithecus* populations isolated in forest fragments (Irwin et al. 2009). If *C. spelea* exhibited analogous predation behavior, it is plausible that subfossil lemurs living in habitats that were contracting as a result of anthropogenic activity may have been at increased risk of terminal exploitation by both extant and giant fossa. Extinct avian predators are represented by two species of *Aquilla*, and one species of *Stephaneatus*; the larger of the genus *Aquilla* was approximate in size to the extant golden eagle (*Aquilla chryseatus*) at 3–6 kg, which are fierce predators known to pursue large mammals several times their size, up to 70 kg (Kerley and Slaght 2013). The extant member of genus *Stephaneatus* in continental Africa (slightly smaller than the extinct congener of Madagascar) regularly kills primates up to 12 kg (Goodman 1994). It seems tenable that large raptors may have also imposed significant predation pressure on many of the subfossil lemurs after factoring reduced BM estimates for these primates. Additional predation pressure undoubtedly came from the extinct Malagasy crocodilian (*Voahy robustus*); with an estimated adult body length of 3 m (Brochu 2007), this reptile would have been capable of significant predation of all subfossil lemurs, as extant crocodile species of similar size are known to attack and subdue large mammals weighing over 100 kg (Guggisberg 1972). A multitaxa analysis of subfossil lemur bone deformation linked to predatory behavior shows that all represented lemur species had evidence of crocodile predation (Meador 2017). These points taken into consideration of the revised BM estimates demonstrate that we cannot overlook the role that predation by natural predators may have played in contributing to declines in populations of subfossil lemurs, perhaps intensified by a combination of factors originating from or including human activity.

## 7 Conclusion

This study implemented statistical corrections for phylogenetic relatedness in deriving body size estimates for subfossil lemurs. The new body size estimates generated in this study suggest that subfossil lemurs are consistently smaller than the estimates put forth by previous authors, including Jungers et al. (2008). Nonetheless, the largest of these species are still “giant.” That is, *Archaeoindris* (~127 kg) would have been larger than extant adult female gorillas (e.g., *Gorilla beringei* , 97.5 kg), and *M. grandidieri* (~56 kg) would also have been within the range of living male chimpanzees (42.7–59.7 kg; Smith and Jungers 1997). Likewise, *Pachylemur insignis* (~8 kg) *and Pachylemur jullyi* (~10 kg) are still larger bodies than their extant counterparts (*Varecia* spp.) even after accounting for phylogenetic relatedness. However, the body weight of the smallest species in our sample, *Mesopropithecus globiceps* (as well as some species of *Pachylemur*), now overlaps with that of the largest extant lemurs, *Indri indri* and *Propithecus diedema*. Future studies of subfossil lemur life history traits, ecology, and hypotheses concerning anthropogenic extinction should consider these new body size estimates.

## Author Contributions

**Katharine E. T. Thompson:** conceptualization (equal), data curation (equal), formal analysis (equal), funding acquisition (equal), investigation (equal), methodology (equal), visualization (equal), writing – original draft (equal), writing – review and editing (equal). **Ryan S. Rothman:** formal analysis (equal), methodology (equal), writing – original draft (equal), writing – review and editing (equal). **John D. Polk:** data curation (equal), writing – original draft (equal), writing – review and editing (equal). **F. Tré Lawrence:** writing – original draft (equal), writing – review and editing (equal). **William Jungers:** conceptualization (equal), project administration (equal), supervision (equal). **Gabrielle A. Russo:** project administration (equal), supervision (equal), writing – original draft (equal), writing – review and editing (equal).

## Supporting information


**Figure S1:** Phylogenetic tree of 62 extant primate species, reflecting the taxa which were used to predict subfossil body mass. Scale bar represents evolutionary divergence time in millions of years.

**Table S1:** Data sources for each extinct and extant species used in this study. Extinct species sources include Jungers et al. (2008) and original data, while extant species sources include works by Demes, Jungers, Polk, and Runestad.

## Data Availability

Data Availability Statement: The data that support the findings of this study are available from the corresponding author upon reasonable request.
