# Supplementary figures and images for: Revised Body Mass Estimates for Extinct Lemurs
Source: Am J Biol Anthropol. 2025 Nov 18;188(3):e70158. doi: 10.1002/ajpa.70158 (PMC12625801; doi:10.1002/ajpa.70158)

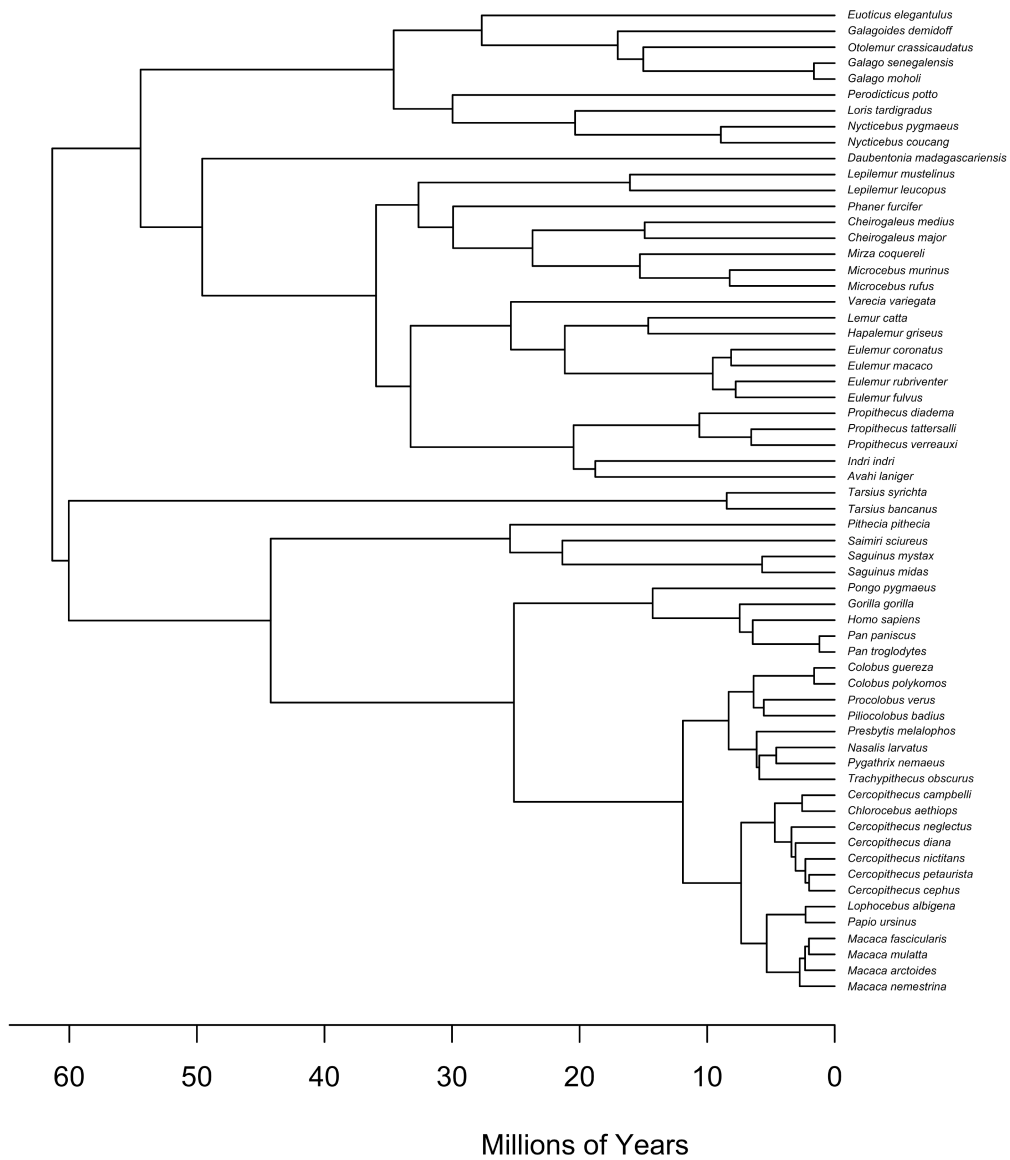

Supplement: Supplementary file 1 — Figure S1: Phylogenetic tree of 62 extant primate species, reflecting the taxa which were used to predict subfossil body mass. Scale bar represents evolutionary divergence time in millions of years. [file AJPA-188-e70158-s001.pdf]
