# Supplementary material for: Revised Body Mass Estimates for Extinct Lemurs
Source: Am J Biol Anthropol. 2025 Nov 18;188(3):e70158. doi: 10.1002/ajpa.70158 (PMC12625801; doi:10.1002/ajpa.70158)
Supplement: Supplementary file 2 — Table S1: Data sources for each extinct and extant species used in this study. Extinct species sources include Jungers et al. (2008) and original data, while extant species sources include works by Demes, Jungers, Polk, and Runestad. [file AJPA-188-e70158-s002.docx]

| **Extinct** |  |
| --- | --- |
| **Species** | **Source(s)** |
| Archaeolemur edwardsi | Thompson et al.; Jungers et al. (2008) |
| Archaeolemur majori | Thompson et al.; Jungers et al. (2008) |
| Megaladapis edwardsi | Thompson et al.; Jungers et al. (2008) |
| Megaladapis grandidieri | Thompson et al.; Jungers et al. (2008) |
| Megaladapis madagascariensis | Thompson et al.; Jungers et al. (2008) |
| Mesopropithecus globiceps | Thompson et al.; Jungers et al. (2008) |
| Mesopropithecus pithecoides | Thompson et al. |
| Pachylemur insignis | Thompson et al.; Jungers et al. (2008) |
| Palaeopropithecus ingens | Thompson et al.; Jungers et al. (2008) |
| Palaeopropithecus maximus | Thompson et al.; Jungers et al. (2008) |
| Archaeoindris fontoynontii | Jungers et al. (2008) |
| Babakotia radofilai | Jungers et al. (2008) |
| Daubentonia robustus | Jungers et al. (2008) |
| Mesopropithecus dolichobrachion | Jungers et al. (2008) |
| Pachylemur jullyi | Jungers et al. (2008) |
| Archaeolemur sp. | Jungers et al. (2008) |
| **Extant** |  |
| **Species** | **Source(s)** |
| Avahi laniger | Demes and Jungers (1993) |
| Cercopithecus campbelli | Polk (unpublished); Smaers et al. (2021) |
| Cercopithecus cephus | Polk et al. (2000) |
| Cercopithecus diana | Polk et al. (2000) |
| Cercopithecus neglectus | Polk et al. (2000) |
| Cercopithecus nictitans | Polk et al. (2000) |
| Cercopithecus petaurista | Polk et al. (2000) |
| Cheirogaleus major | Runestad (1994) |
| Cheirogaleus medius | Demes and Jungers (1993) |
| Chlorocebus aethiops | Polk et al. (2000) |
| Colobus guereza | Polk et al. (2000) |
| Colobus polykomos | Polk (unpublished); Smaers et al. (2021) |
| Daubentonia madagascariensis | Runestad (1994) |
| Eulemur coronatus | Runestad (1994) |
| Eulemur fulvus | Demes and Jungers (1993) |
| Eulemur macaco | Runestad (1994) |
| Eulemur rubriventer | Runestad (1994) |
| Euoticus elegantulus | Demes and Jungers (1993) |
| Galago moholi | Demes and Jungers (1993) |
| Galago senegalensis | Polk et al. (2000) |
| Galagoides demidoff | Runestad (1994) |
| Gorilla gorilla | Polk et al. (2000); Carlson (2005) |
| Hapalemur griseus | Runestad (1994) |
| Homo sapiens | Carlson (2005) |
| Indri indri | Demes and Jungers (1993) |
| Lemur catta | Demes and Jungers (1993) |
| Lepilemur leucopus | Demes and Jungers (1993) |
| Lepilemur mustelinus | Runestad (1994) |
| Lophocebus albigena | Polk et al. (2000) |
| Loris tardigradus | Demes and Jungers (1993) |
| Macaca arctoides | Polk et al. (2000) |
| Macaca fascicularis | Polk et al. (2000) |
| Macaca mulatta | Polk et al. (2000) |
| Macaca nemestrina | Polk et al. (2000) |
| Microcebus murinus | Demes and Jungers (1993); Carlson (2005) |
| Microcebus rufus | Runestad (1994); Carlson (2005) |
| Mirza coquereli | Demes and Jungers (1993) |
| Nasalis larvatus | Polk et al. (2000) |
| Nycticebus coucang | Runestad (1994) |
| Nycticebus pygmaeus | Runestad (1994) |
| Otolemur crassicaudatus | Runestad (1994) |
| Pan paniscus | Carlson (2005) |
| Pan troglodytes | Polk et al. (2000); Carlson (2005) |
| Papio ursinus | Polk et al. (2000) |
| Perodicticus potto | Runestad (1994) |
| Phaner furcifer | Runestad (1994) |
| Piliocolobus badius | Polk et al. (2000) |
| Pithecia pithecia | Runestad (1994) |
| Pongo pygmaeus | Polk et al. (2000) |
| Presbytis melalophos | Polk et al. (2000) |
| Procolobus verus | Polk et al. (2000) |
| Propithecus diadema | Demes and Jungers (1993) |
| Propithecus tattersalli | Demes and Jungers (1993) |
| Propithecus verreauxi | Demes and Jungers (1993) |
| Pygathrix nemaeus | Polk et al. (2000) |
| Saguinus midas | Runestad (1994) |
| Saguinus mystax | Runestad (1994) |
| Saimiri sciureus | Runestad (1994) |
| Tarsius bancanus | Runestad (1994) |
| Tarsius syrichta | Runestad (1994) |
| Trachypithecus obscurus | Polk et al. (2000) |
| Varecia variegata | Demes and Jungers (1993) |
